# Evaluating the Performance and Stability of the Highway Subgrades in Seismic Events, a Case Study of the Changning Earthquake, Sichuan

**DOI:** 10.3390/ijerph192114379

**Published:** 2022-11-03

**Authors:** Zhen Cui, Maochu Zhang, Kai Wu, Hongsheng Ma

**Affiliations:** 1 State Key Laboratory of Geomechanics and Geotechnical Engineering, Institute of Rock and Soil Mechanics, Chinese Academy of Sciences, Wuhan 430071, China; 2 School of Engineering Science, University of Chinese Academy of Sciences, Beijing 100049, China; 3 Key Laboratory of Changjiang Regulation and Protection of Ministry of Water Resources, Changjiang Engineering Group, Wuhan 430010, China; 4 Sichuan Highway Planning, Survey, Design and Research Institute Ltd., Chengdu 610041, China

**Keywords:** earthquake, fill subgrade, seismic damage mode, relative displacement, dynamic response

## Abstract

On 17 June 2019, an M6.0 earthquake occurred in Changning County, Sichuan Province, China. Considerable highway subgrades were damaged in this earthquake. By investigating seismic damage of these subgrades, a systematical analysis of influence factors and failure mode of the damages on highway subgrade have been given. There is a close relationship between the damaged degree of subgrade and the distance of epicenter, fault distance, and structure type of subgrade. The seismic hazard investigation shows that the seismic damage of the cut-and-fill subgrade was more severe than that of the fill subgrade. Taking the Changning earthquake ground motion record as input, 3D dynamic finite element analyses were performed. The seismic damage mechanisms of cut-and-fill subgrade and fill subgrade were revealed. The numerical simulation confirmed that the cut-and-fill subgrade was more easily damaged by earthquakes compared with the fill subgrade. The fill-and-cut interface of the cut-and-fill subgrade had a notable plastic strain band after the earthquake, and the permanent displacement of the slope top was significant. In this manner, the numerical results are consistent with seismic investigation data. Considering the seismic investigation data for highway subgrades are rare, this paper may provide effective references for aseismic design and deformation control of highway subgrades.

## 1. Introduction

**On 17 June** 2019: an M.6.0 earthquake struck Changning County, Yibin City, Sichuan Province, China. The impact of this earthquake piled up with that of four subsequent strong aftershocks (M.5.1, M.5.3, M.5.4, and M.5.6) [1]. According to the seismic intensity map published by China Earthquake Administration, which was made according to the Chinese code for “Post-earthquake field works—Part 3: Code for field survey (GB/T 18208.3-2011)” [2], the seismic intensity in this earthquake was up to level VIII. The earthquake occurred on a seismic fault near Changning-Shuanghe anticline on the edge of the Sichuan Basin in the southeast of Sichuan [3]. Compared with other earthquakes, this earthquake was followed by many aftershocks. The seismic hazard assessment report showed that the disaster-stricken areas mainly included nine counties and districts, including Changning County and Gongxian County in Yibin City, and that the transportation infrastructure in the disaster-stricken areas was damaged to varying degrees. Among them, the Longtou-Shuanghe expressway, about 5 km from Yibin-Xuyong (Yi-Xu) Expressway, was seriously damaged. The seismic damage pattern is mainly expressed as tensile crack-induced deformation of subgrades. Among them, the seismic hazard of the cut-and-fill subgrade was the most obvious.

Historically, seismic events were active in the western mountainous areas of China. Several major earthquakes occurred in this region in recent decades, such as the Wenchuan earthquake (2008), the Lushan earthquake (2013), and the Jiuzhaigou earthquake (2017). These earthquakes caused a lot of damage to highways in these areas. However, in these earthquakes, landslides and rockfalls on the upper slope of the highway were the most frequently reported geological disasters. Although some damage cases were reported in the highway subgrades, the seismic damage of the subgrade has received less attention [4,5,6,7]. Yao et al. [8] investigated seismic damages of 13 embankment sites along the Dujiangyan-Yingxiu country motorway and analyzed the seismic damage modes of embankment works and shoulder walls in the 2008 Wenchuan earthquake. Ji et al. [9] investigated seismic damages of the upper slope engineering and subgrades of highways in the Wenchuan earthquake area and concluded that commonly high and steep embankments have poor seismic performance. Wang et al. [10] analyzed the seismic damage mode of embankment and the performance of geogrids with reinforcement in deformation control in the Wenchuan earthquake area through a shaking table model test and numerical simulation. Refs. [11,12,13] simulated the seismic dynamic response and damage process of fill subgrades through large shaking table model tests. As for the numerical method, Morteza and Hamidreza [14] performed a 3D finite-element simulation for railway subgrades. Zhao [15] analyzed the dynamic response of two types of subgrades, i.e., integrated and separated highways subgrades, by 2D nonlinear dynamic finite element analysis method. A combined simple and advanced numerical procedure was proposed by Gaetano and Mohamed [16] to assess the seismic performance of embankments, and more related works can be found in Rampello et al. [17], Aydingun and Adalier [18], Işık et al. [19], Masini et al. [20], and Bilgin et al. [21]. The above studies have promoted the understanding of the seismic performance of subgrades and the development of the seismic design concept of subgrades.

Currently, most of the existing literature about seismic damage investigation and analysis of subgrades in China is based on common fill (embankment) subgrades of low-level highways such as national and provincial motorways. Limited literature exists for high-level mountain expressway subgrades, especially for cut-and-fill subgrades. In the Changning earthquake, the Yi-Xu Expressway in the area suffered much seismic damage to the subgrades. The type of subgrade is mainly cut-and-fill in the area. In this manner, the experience learned from the performance and stability of these highway subgrades are valuable and representative.

In this paper, seismic performance and stability of the subgrades of the Yi-Xu Expressway in the Changning earthquake were evaluated. Field investigation, dynamic tri-axial test, and dynamic numerical analysis were conducted. The response and seismic damage mechanism of the cut-and-fill subgrades were revealed. The performed work would greatly enrich the data on seismic damage investigation of highway subgrades and provide valuable research results for seismic design and dynamic deformation control of subgrades.

## 2. Seismic Damage Assessment of the Subgrades

### 2.1. Overview of the Yi-Xu Highway Project

The Yi-Xu Expressway is 114.07 km long, starting from Cuiping District, Yibin City, and ending at Xuyong County, Luzhou City, and this expressway project was completed and opened to traffic in June 2016. In this project, chainage K0–K29 and chainage K81–K114 pass through the hilly areas of red beds, and chainage K29–K81 pass through low mountainous areas with tectonic denudation and dissolution. The design-basis peak acceleration at the site is 0.05 g, and the characteristic period of the seismic response spectrum is 0.35 s with a basic design seismic intensity of level Ⅵ.

**Figure 1:**
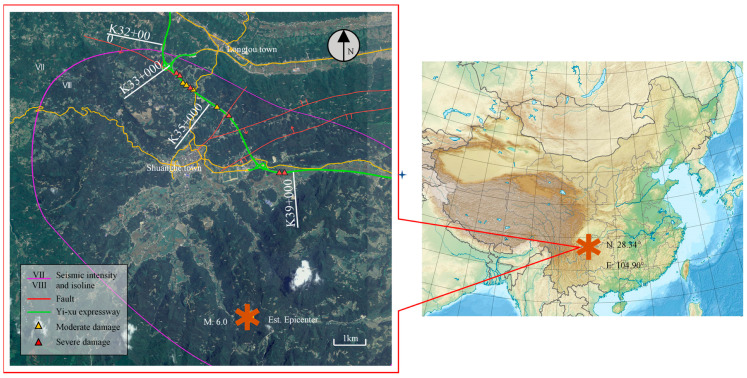
The damaged locations of the highway subgrades in the Changning earthquake event.

An M.6.0 earthquake occurred in Changning on 17 June 2019, with the highest intensity reaching level Ⅷ. The estimated location of the epicenter was marked based on the coordinate according to the public seismic report, as shown in Figure 1. The focal depth is approximately 16 km. The focal mechanism solution shows that it is a strike-slip earthquake. The chainage K33 + 000–K43 + 500 of the Yi-Xu Expressway, about 10.5 km long, was in the level Ⅷ area in this earthquake. As can be noted in Figure 1, at chainage K32 + 000, there was a Dafenba fault that intercourses with the expressway route, whose total length is about 2.1 km. The Jinyutang active fault was developed at chainage K36 + 165, the general striking angle of the fault is 35°, the dip direction is to NW, and the dip angle is approximately 72°. The Jinyutang active fault was nearly perpendicular to the route of the expressway. The total length of the fault was 2.5 km, and the width of the fracture zone varied from 3 to 20 m. At chainage K36 + 800 section, the Xuedangwan fault was developed; the general striking angle of the fault is 75°, the dip direction is to SE, and the dip angle is approximately 85°. The fault intersected the expressway route at a large angle and extended southward to the tip of the Jingyutang fault. The total length of the fault was 7.8 km, and the width of the fracture zone varied from 5 to 30 m.

**Figure 2:**
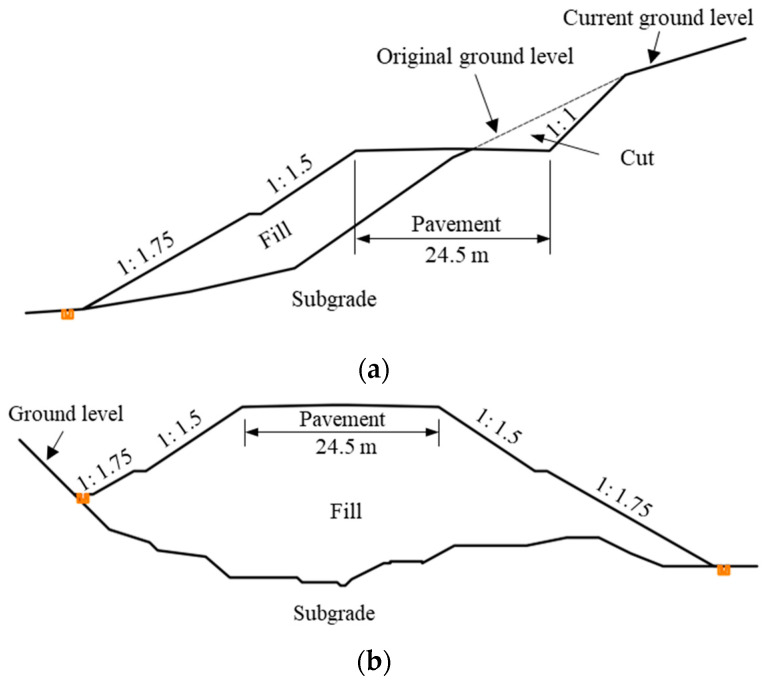
Cross-sections of the typical highway subgrades. (**a**) A typical cut-and-fill section. (**b**) A typical fill section.

At chainage K33 + 000–K39 + 000, the terrain along the route was undulating, and most of them were cut-and-fill subgrades (Figure 2a). The slope ratio of the fill portion is 1:1.5–1:2.0, and the retaining wall is used at the toe for some portions. As a comparison, at chainage K39 + 000–K43 + 500, the terrain was relatively gentle, mostly fill (embankment) subgrades (Figure 2b).

The seismic damage report finished after the Changning earthquake confirmed that almost all subgrade seismic that were damaged were within the K33 + 000–K39 + 000 section, and mainly the cut-and-fill subgrades.

### 2.2. Characteristics of Subgrade Seismic Damages and Influence Factors

As stated above, the subgrades in the area are mainly divided into cut-and-fill subgrades and fill subgrades. Among them, subgrades with a maximum fill height of fewer than 10 m were not damaged by the earthquake, so sites with a fill height of more than 10 m in this affected section were selected for detailed investigation and data analysis. As shown in Table 1, there were 18 sites that were damaged by the earthquake, including 10 fill subgrades and 8 cut-and-fill subgrades. Characteristics of seismic damages to subgrades and influence rules are as follows.

(a) Influence of the epicenter distance and seismic intensity

The seismic intensity was the main factor affecting the degree of development of highway seismic damages, as shown in Figure 1. According to field investigation statistics, highway subgrades in the level VII area did not suffer from damages, all seismic damage sites of subgrades were in the level VIII area, and the distance from the epicenter was within 4–7 km.

(b) Influence of distance to fault

As shown in Figure 1, the main faults along the route were Jinyutang, Xuetangwan, and Dafenba. According to the distribution of the seismically damaged subgrade sites, the distance between seismic damage sites and faults was less than 2 km, and subgrade sites far away from faults basically did not show obvious seismic damage.

(c) Influence of subgrade type

As shown in Table 1, the investigation of subgrade seismic damages showed that the fill height of subgrades had little effect on the seismic performance of subgrades, while the type of subgrades can significantly affect the seismic performance. Although the maximum height of a fill subgrade was up to 21.6 m, its seismic damage was minor. However, among the eight cut-and-fill subgrades, although the fill height of some subgrades was less than 15 m, there were still six subgrades with severe damage, with the original ground slope angle of these subgrades all above 22°. The preliminary analysis of cut-and-fill subgrades showed that since seismic damages were mainly due to the difference in geotechnical properties between fill and cut portions, the inconsistency of hardness and compactness of the base course, and steep original ground slopes. When an earthquake occurred, it would be easy to slide along the fill-and-cut interface or the geotechnical interface, resulting in deformation, cracking, and sliding failure of subgrades.

### 2.3. Seismic Damage Mode of Subgrades

**Figure 3:**
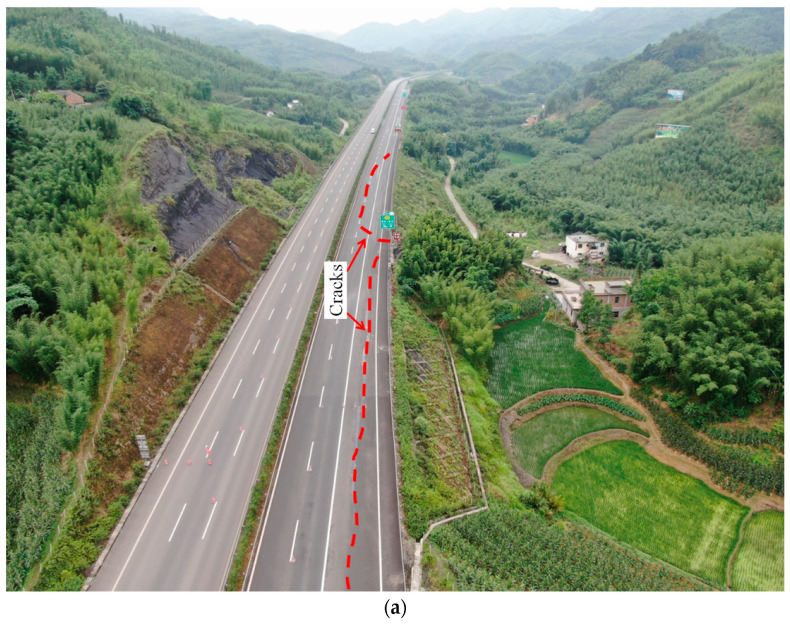

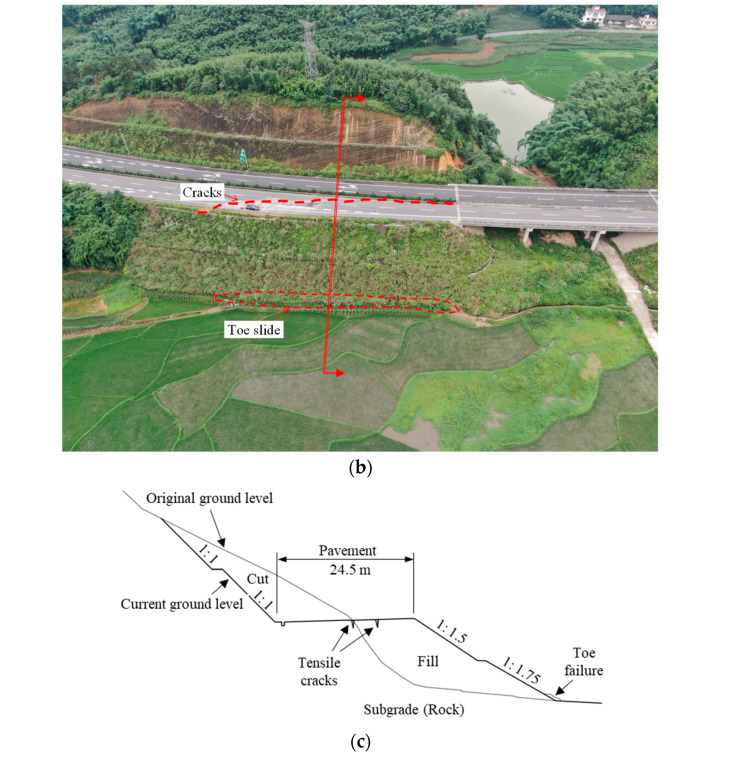
Photo of the typical seismic damage mode of the cut-and-fill sections of chainage K33 + 000-K39 + 000. (**a**) (K34 + 050~K34 + 350). (**b**) K38 + 550~K38 + 750. (**c**) Cross-section of K38 + 650.

In this earthquake, seismic damages of the cut-and-fill subgrade were obvious. As shown in Figure 3, taking the subgrade of K38 + 550–K38 + 750 section as an example, the maximum fill height was 17.4 m, the fill height of primary slopes was 8 m with a slope ratio of 1:1.5, and the fill height of secondary slopes was 12 m with a slope ratio of 1:1.75. The fill slopes were reinforced with diamond-shaped framework structures. The basic design seismic intensity of this road section in design was only level VI. However, on 17 June 2019, the seismic intensity of this site in the Changning earthquake reached level VIII. As shown in Figure 3c, under the influence of the earthquake, tensile cracks appeared along the road surface at the fill-and-cut interface. The width of the cracks was about 6–20 cm and cracks extended about 200 m. The overall subsidence of subgrades was about 30 cm, and there were 5–10 cm down-staggered cracks on the outer side of the pavement. Under the coupling effect of seismic force and gravity, the slope toe of the embankments was bulging and deformed, and the diamond-shaped framework structures and the ditch at the slope toe were deformed and cracked due to squeezing action. From the perspective of the overall distribution of cracks, the earthquake led to deformation characteristics of half subgrades, such as the upper part cracking and the lower part bulging, and there was a risk of sliding.

Seismic damages of cut-and-fill subgrades in this earthquake indicated that the deformation and damages of subgrades under earthquake were mainly the action process of horizontal seismic inertia force. The existing shaking table test also verified such phenomenon [22], that is, the subgrade topography can amplify input seismic waves, and the amplification effect is most obvious near the pavement shoulder. Therefore, the top of the fill subgrade is the weakest point, and it is easy to deform and crack first in an earthquake. After the crack at the fill-and-cut interface runs through the bulging and cracks at the slope toe, it is easy to lead to overall instability. This mode is a typical mode of instability and failure of cut-and-fill subgrades under earthquake action.

## 3. Dynamic Test of Subgrade Soil Characteristics

### 3.1. Dynamic Test Setup

Before the numerical simulation of the seismic damage mechanism of subgrades, the dynamic test was started to obtain the dynamic parameters of subgrade soils so as to carry out 3D dynamic numerical simulation.

**Figure 4:**
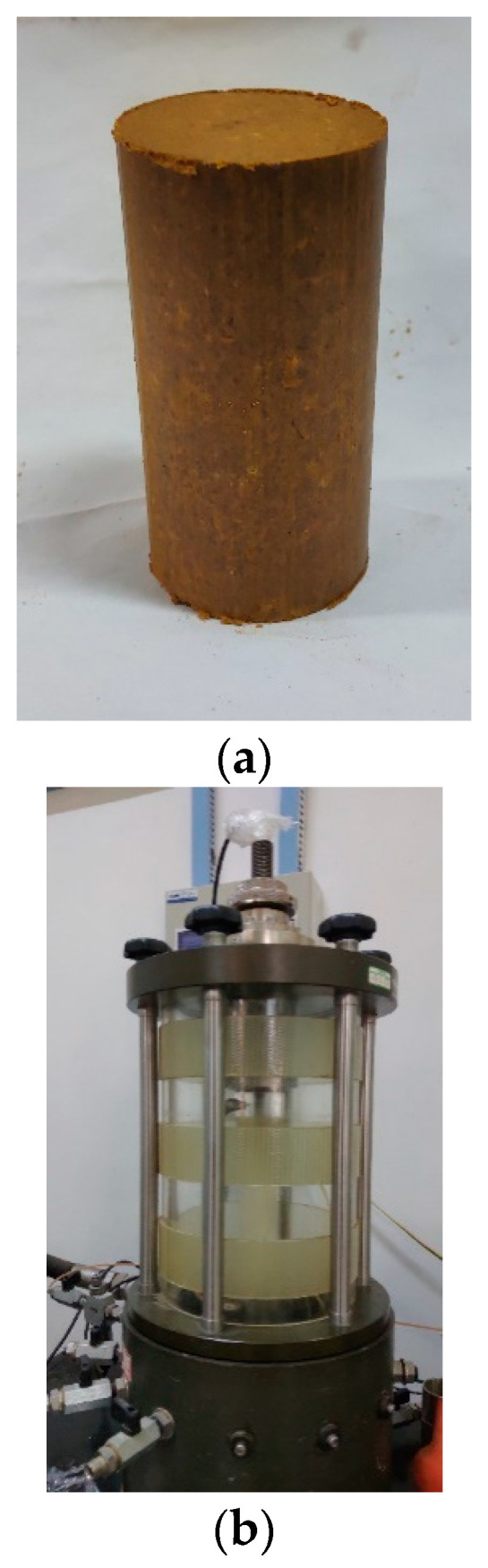
Dynamic triaxial test of silty clay. (**a**) Silty clay specimen. (**b**) GDS dynamic triaxial test system.

Therefore, silty clay samples of fill subgrades were obtained from K38 + 550–K38 + 750 section and prepared into test specimens. Remodeled samples were prepared; that is, the collected soil samples were dried, ground, and sieved to prepare bulk soil with a water content of 20%, which was kept in an airtight and non-evaporating environment for 6 h to make the soiled uniform, and then samples with a diameter of 38 mm and a height of 76 mm were molded (Figure 4a). The cyclic loading test was carried out on the GDS dynamic triaxial test system with variable confining pressure (Figure 4b). The dynamic cyclic mode with stress control was selected; the target confining pressures were set at 50 kPa, 100 kPa, and 150 kPa; the frequency was set at 1 Hz; a half-sine wave was chosen. Then, after setting the amplitude of dynamic stress and vibration cycles, dynamic mechanical parameters of silty clay samples were obtained.

### 3.2. Dynamic Test Results

**Figure 5:**
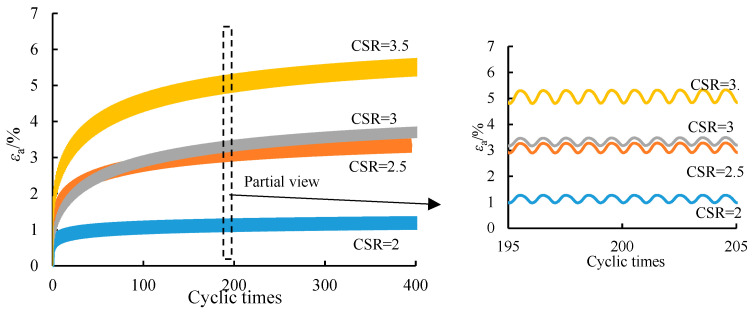
Axial cumulative strain curve of soil samples with various CSR at 100 kPa confining pressure.

In the test, the cyclic load amplitude mainly played its role by controlling the cyclic stress ratio (*CSR*), which was calculated according to the following formula. Figure 5 shows the development curve of axial strain when the *CSR* was 2, 2.5, 3, and 3.5, respectively, under a confining pressure of 100 kPa when the number of cycles was 400.

(1) $$CSR=\frac{q}{2p^{\prime }}$$

where *q* is the amplitude of cyclic shear stress and *p*′ is the initial effective confining pressure.

**Figure 6:**
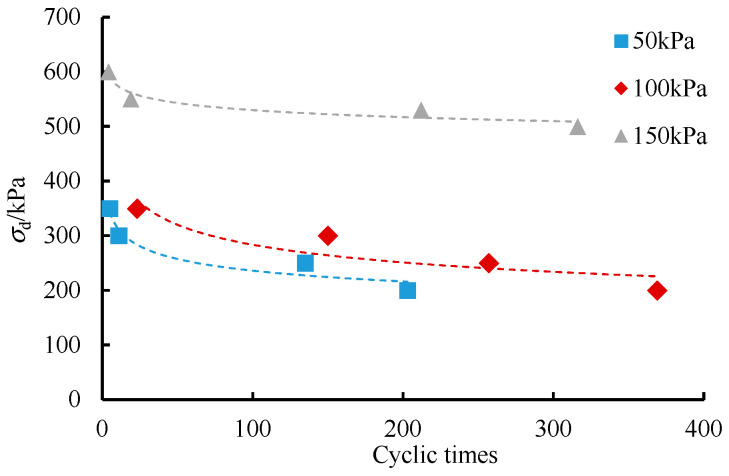
Dynamic strength–critical cycle number under various confining pressures.

Under the condition of constant frequency and vibration cycles, a cumulative strain of 3% was deemed as the criterion to judge whether damages occurred, and the dynamic strength was characterized by the amplitude of dynamic stress. According to the test results, the vibration cycles required when the accumulated strain of soil samples reached 3% (critical cycle number) under different confining pressures were determined, and the relationship curve between the amplitude of samples and failure vibration times under different confining pressures was drawn, as shown in Figure 6.

The test results showed that the subgrade soil shows the following characteristics under cyclic loading: (1) the smaller the amplitude, the more vibration cycles are required for samples to achieve failure; (2) the larger the confining pressure, the greater the lateral deformation restriction force of soil samples, and the stronger the friction and interlocking between particles, thus increasing the vibration cycles required for the failure of the soil samples.

### 3.3. Calculation of Dynamic Strength

Considering the effects of confining pressure, amplitude, and vibration cycles on the failure of silty clay samples in this test, the relationship between *CSR* and failure vibration cycles *N* can be represented by a power function, and the following results can be obtained:

(2) $$CSR=aN^{-b}$$

where *N* is the failure vibration cycles, and a and b are fitting parameters.

According to *CSR*-*N* expression under various confining pressures, the axial dynamic deviator stress under certain confining pressures and vibration cycles was calculated, and then the slots of Mohr’s circle and strength envelope under different failure vibration times were obtained. According to Mohr’s circle and strength envelope, the dynamic mechanical parameters under different vibration times were obtained, as shown in Table 2.

It can be found that the dynamic strength of soil samples is greatly influenced by the confining pressure and vibration cycles and that with the increase in confining pressure and vibration cycles, the cohesion of soil samples increases and the internal friction angle decreases.

## 4. Dynamic Response Analysis of Subgrades

To reveal the seismic damage mechanism of the two types of subgrades, the characteristics of seismic dynamic response were numerically simulated.

### 4.1. Dynamic Simulation Conditions

**Figure 7:**
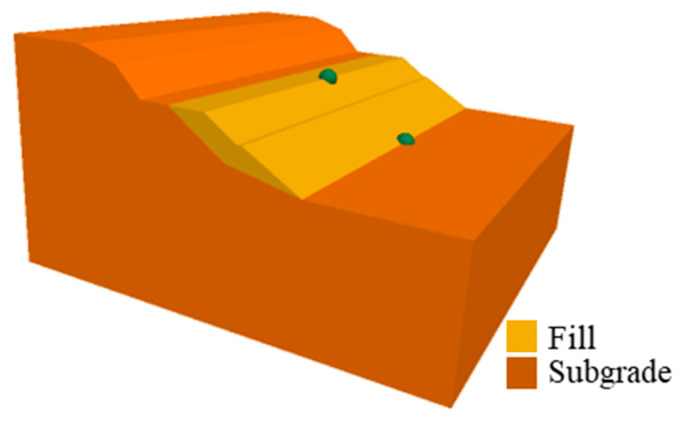
Numerical model and numerical monitoring points of the cut-and-fill subgrade.

**Figure 8:**
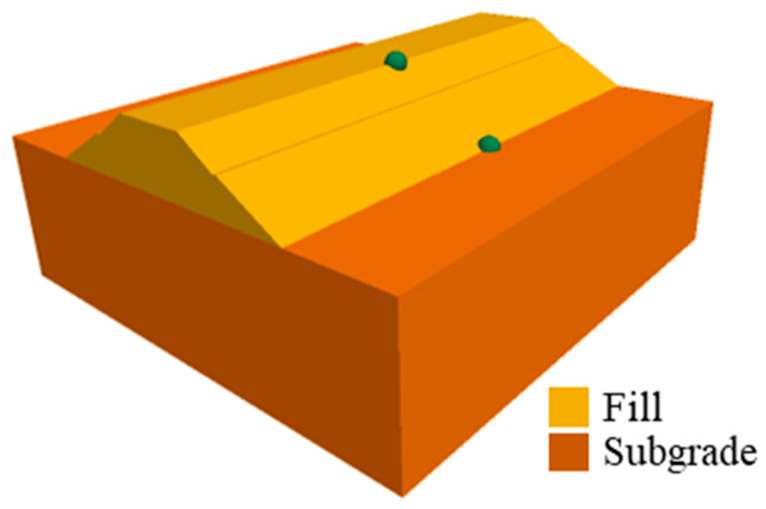
Numerical model and numerical monitoring points of the fill subgrade.

According to the seismic damage mode mentioned above, the results were analyzed with the full time-history dynamic simulation method. Two types of subgrades were considered, i.e., the cut-and-fill type and the fill type. The 3D numerical models of these two kinds of subgrade were established in FLAC3D, a 3D FDM software. As shown in Figure 7 and Figure 8, during the numerical simulation of seismic dynamics of these two kinds of subgrades, all conditions, such as material parameters and seismic action, were kept consistent.

The FDM model was 80 m in length and width. The model top is set to be free, and the seismic waves are applied to the bottom of the model during numerical simulations, and a free-field boundary condition was utilized for the lateral boundaries. In dynamic analysis, both the frequency content of the input wave and the wave speed characteristics of the system will affect the numerical accuracy of wave transmission. Kuhlemeyer and Lysmer [23] show that for an accurate representation of wave transmission through a model, the spatial element size must be smaller than from approximately one-tenth to one-eighth of the wavelength associated with the highest frequency component of the input wave, as expressed in Equation (3)

(3) $$∆l\leq \left(\frac{1}{8}\sim \frac{1}{10}\right)L$$

where L is the wavelength associated with the highest frequency component for peak velocities through the medium. In this study, a fine mesh with a maximum element size of 3~5 m was adopted.

In the free-field boundary technique, lateral boundaries of the main grid are coupled to the free-field grid by viscous dashpots to simulate a quiet boundary, and the unbalanced forces from the free-field grid are applied to the main grid boundary. Both conditions are expressed in Equations (4)–(6), which apply to the free-field boundary along one side boundary plane with its normal in the direction of the x-axis. Similar expressions may be written for the other sides and corner boundaries:

(4) $$F_{x}=-\rho C_{p}\left(v_{x}^{m}-v_{x}^{ff}\right)A+F_{x}^{ff}$$

(5) $$F_{y}=-\rho C_{s}\left(v_{y}^{m}-v_{y}^{ff}\right)A+F_{y}^{ff}$$

(6) $$F_{x}=-\rho C_{s}\left(v_{z}^{m}-v_{z}^{ff}\right)A+F_{z}^{ff}$$

where ρ is the density of rock mass, $C_{p}$ is the P-wave velocity at the model boundary, $C_{s}$ is S-wave velocity at the model boundary, A is the area of influence of a free-field grid point, $v_{i}^{m}$ is the i-direction velocity of a grid point in the main grid at the model boundary, $v_{i}^{ff}$ is the i-direction velocity of a grid point in a side free field, $F_{i}^{ff}$ is the free-field grid point force in i-direction.

Rayleigh damping [23] was used. A range of 2~5% for the damping factor of geological materials was suggested by Biggs [24], and a moderate value of 3% was used in the current study.

Based on the results of the geotechnical engineering classification and indoor dynamic mechanical tests above, the material parameters for numerical simulation were obtained, as shown in Table 3. Since the duration of the earthquake is about 1 min, the dynamic parameters with 50 times of vibration were selected for calculation. In order to reveal the inhomogeneity of geotechnical materials, the point-estimation randomized technique was adopted in numerical simulation. According to the average values in the table, it was considered that the material parameters met the Gaussian distribution so as to make the numerical simulation results closer to actual situations. A careful distribution range is assumed, and the variance of all parameters is 10% of their mean value.

**Figure 9:**
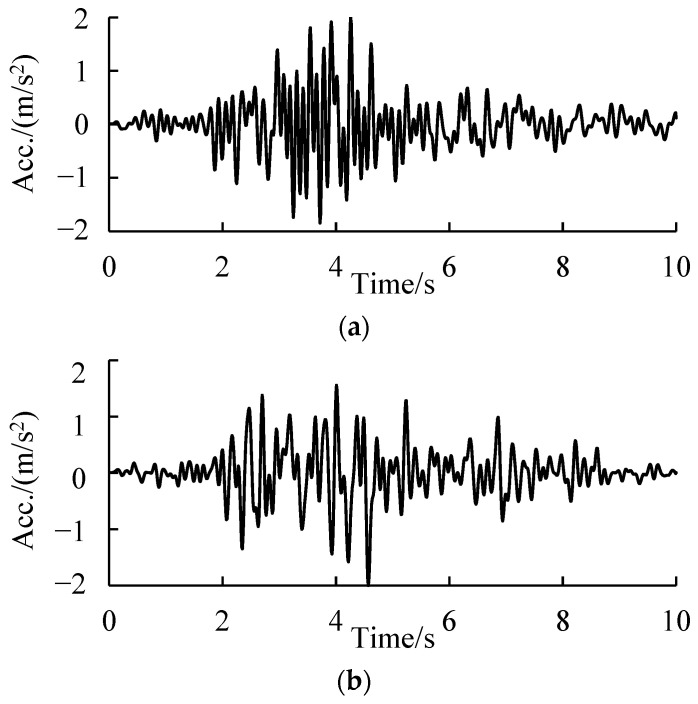
Acceleration time–history curve of the earthquake used in the numerical simulation. (**a**) Direction of perpendicular to the fault. (**b**) Direction of Parallel to the fault.

As for the seismic motions, the measured time history records of the Changning earthquake were collected from a strong earthquake database (Figure 9). After being broken down according to directions, they were input into the numerical model. According to the intensity map shown in Figure 1, the input intensity of the ground motions was set to be 0.15 g, which corresponds to the level VIII intensity.

### 4.2. Seismic Dynamic Response

The following section discusses the seismic dynamic response characteristics based on these two subgrade models.

(1) Cut-and-fill subgrades

**Figure 10:**
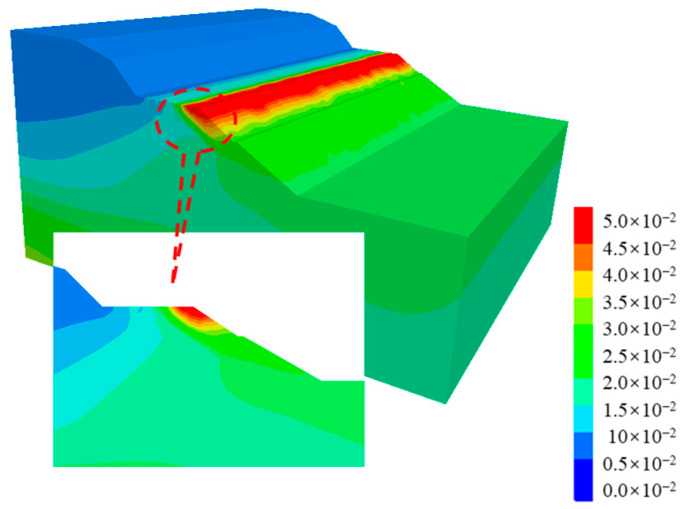
Displacement contour of the cut-and-fill subgrade after earthquake.

**Figure 11:**
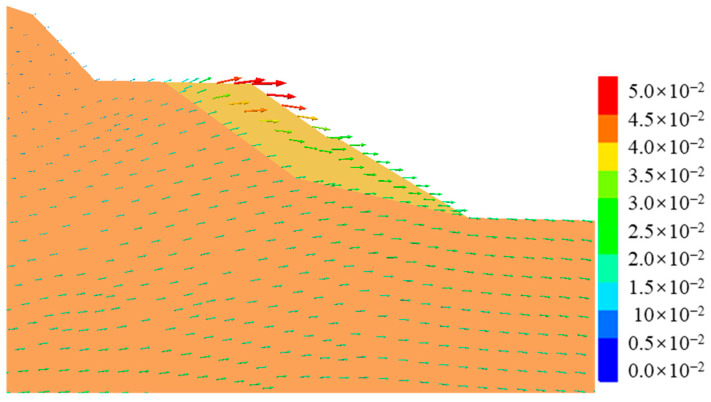
Displacement vectors of the cut-and-fill subgrade after earthquake.

Figure 10 shows the post-earthquake displacement contour of cut-and-fill subgrades. It can be seen from Figure 10 that the post-earthquake displacement mainly occurred in the middle of the shoulder filling, that the maximum displacement value was about 5 cm, and that there was also a small bulging deformation, about 2–3 cm. Figure 11 shows the displacement vector diagram in the middle section of the numerical model. It can be seen from the figure that the post-earthquake deformation mainly occurred on the filling side of subgrades, extending horizontally from the upper part of the subgrade to the outside of the slope and that the lower part showed a tendency of extrusion and bulging. Seismic-induced dynamic shear stress led to tensile cracks in the pavement, while dynamic soil pressure led to the bulging and deformation of the lower part of the subgrade. The post-earthquake displacement trend of subgrade in Figure 10 and Figure 11 was essentially consistent with actual seismic damage investigation results.

**Figure 12:**
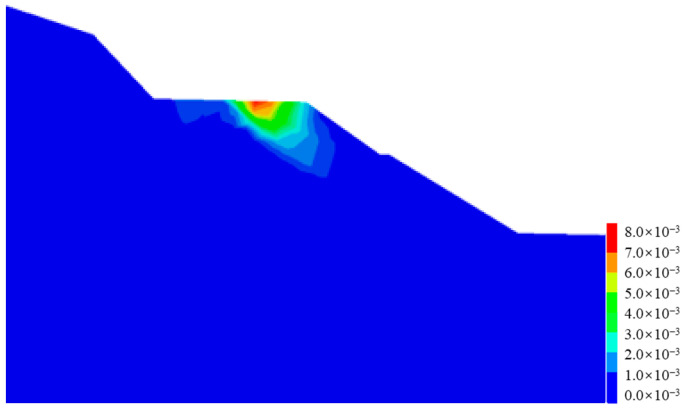
Plastic strain contour of the cut-and-fill subgrade after earthquake.

Figure 12 shows the plastic strain contour of the cut-and-fill subgrade after the earthquake. It can be clearly seen that the post-earthquake plastic strain of the subgrade mainly occurred along the fill-and-cut interface, which can explain why there were arc cracks along the fill-and-cut interface in this section of pavement in the field investigation.

**Figure 13:**
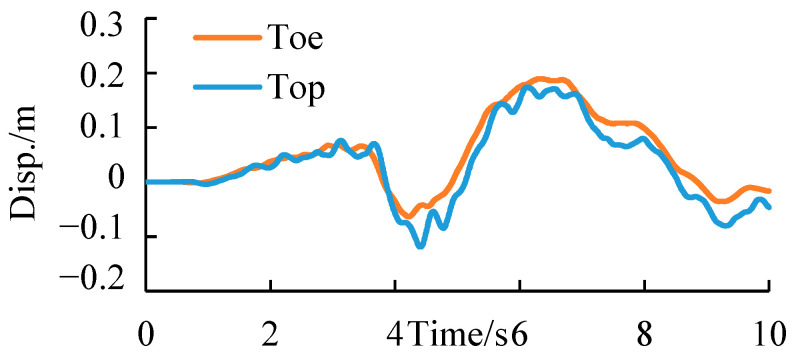
Displacement time–history curves of the monitoring points of the cut-and-fill subgrade.

Figure 13 shows the displacement time-history curve of two monitoring points at the slope toe and top in the cut-and-fill subgrade model. See the green balls in Figure 7 for the layout of monitoring points. According to the displacement time-history curve, the seismic displacement trend at the slope top was similar to that at the slope toe, but the final deformation of the monitoring point at the slope top was about 5 cm after the earthquake.

(2) Fill subgrades

**Figure 14:**
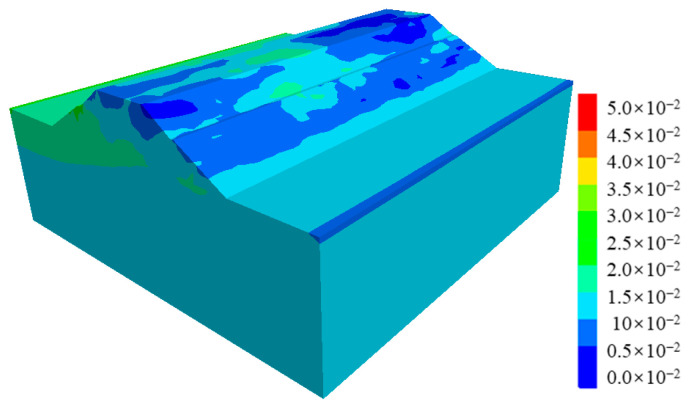
Displacement contour of the fill subgrade after earthquake.

**Figure 15:**
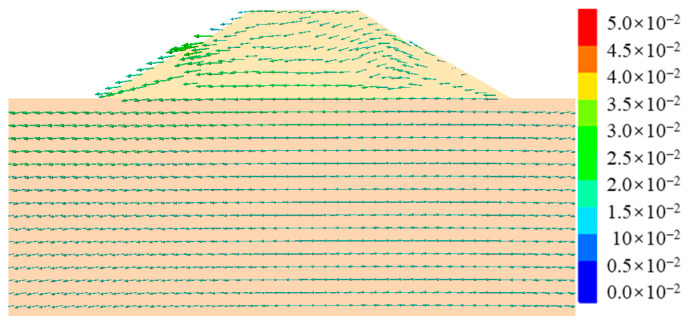
Displacement vectors of the fill subgrade after earthquake.

In order to compare with cut-and-fill subgrades, the seismic deformation and failure mechanism of fill subgrades were given. Figure 14 and Figure 15 are the post-earthquake displacement contour and displacement vector diagrams of fill subgrades, respectively. It can be learned that the post-earthquake deformation of fill subgrades was merely about 1–3 cm compared with that of cut-and-fill subgrades and that deformation extended out of the slope from both sides. Displacement results were basically consistent with unobvious seismic damages of the fill subgrades found in the seismic damage investigation.

**Figure 16:**
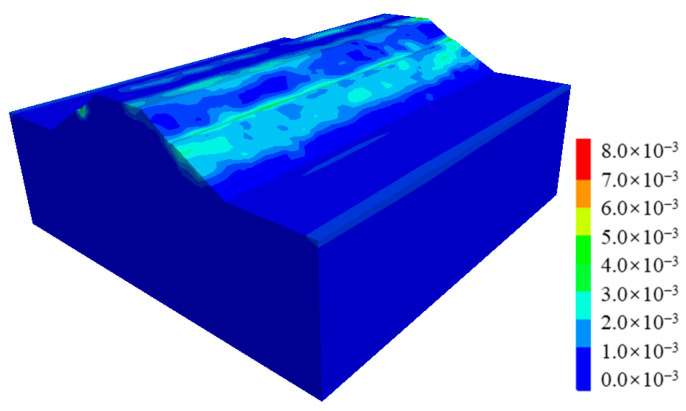
Plastic strain contour of the fill subgrade after earthquake.

Similarly, compared with cut-and-fill subgrades, the post-earthquake plastic strain of fill subgrades was smaller (as shown in Figure 16), the maximum value was only about one-third of that of the cut-and-fill subgrade, and there was no obvious plastic strain concentration zone.

**Figure 17:**
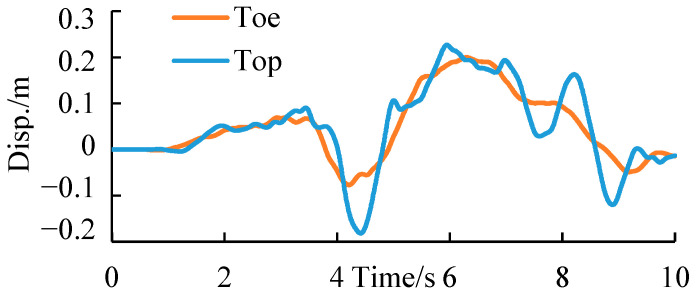
Displacement time–history curves of the monitoring point of the fill subgrade.

Figure 17 shows the displacement time–history curve of two monitoring points at the slope toe and top in the fill subgrade model. The displacement time history at the slope toe was similar to that of the steep-slope model, while the fluctuation of the monitoring point at the slope top was larger, even though the final deformation was relatively small, less than 1 cm. A possible reason may be that under the earthquake, the whole embankment has a strong overall elastic deformation, with large instantaneous deformation and small participating deformation.

**Figure 18:**
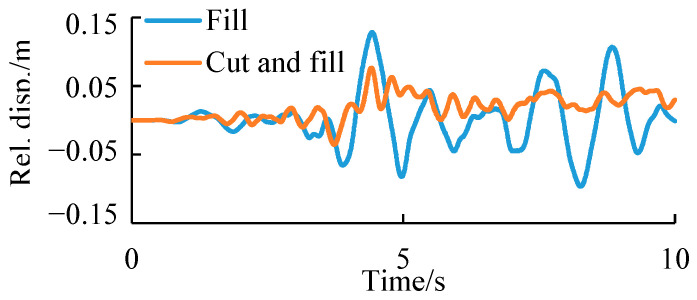
Displacement time-history curves of the relative displacement of top and toe for the two types of subgrades.

In particular, Figure 18 shows the comparison of time-history curves of the relative deformation between the monitoring points at the slope top and toe of these two kinds of subgrades. Comparison results in the figure confirmed the above speculation, that is, when an earthquake occurs, the instantaneous deformation of two-sided fill subgrades is larger, but the post-earthquake permanent deformation is smaller, indicating that its deformation is mainly elastic.

The comparison of seismic numerical simulation between the two kinds of subgrade models showed that under the same seismic conditions, compared with the fill subgrade, the cut-and-fill subgrade was more susceptible to earthquakes. Main seismic damages included large plastic strain along the fill-and-cut interface, large deformation extending from the slope top to outside of the slope, and certain bulging trends at the slope toe, all of which finally led to cracks along the fill-and-cut interface.

## 5. Discussion

According to the investigation of highway damage in the Wenchuan earthquake area, the reinforcement of high-fill subgrades and cut-and-fill with geogrids can significantly reduce the amplification effect of the soil layer by the incident seismic waves. The shaking table model test results of some reinforced embankments [25] also showed that the interaction between geogrids with reinforcement and soil interface can limit the lateral deformation of subgrade filling, increase the natural vibration frequency of subgrades, make the main frequency of external excitation load far away from the resonance frequency band of the subgrades, inhibit the amplification effect of acceleration, and thus reduce the dynamic response amplitude of subgrades. Therefore, for cut-and-fill subgrades in high seismic risk regions, it is recommended to adopt the deformation control with geogrid reinforcement, which focuses on the control of lateral deformation, and geogrids should be laid at the top to improve the seismic performance of subgrades.

## 6. Conclusions

In this paper, influence factors, failure modes, and response characteristics of highway subgrade damages under strong earthquakes are studied by analyzing the actual seismic investigation results and 3D dynamic numerical calculation. The main conclusions are as follows:

(1) The degree of damage to the highway subgrade under the effect of a strong earthquake is related to the distance from the epicenter, fault layout, and type of the subgrade. In the Changning earthquake, highway subgrades in the area of level VII intensity did not suffer from damage; all seismic damage sites of subgrades were in the area of level VIII intensity and near faults, and the distance from the epicenter was within 4–7 km. There is no obvious relationship between the degree of seismic damages of subgrades and the fill height, but the degree of damages is greatly influenced by the type of subgrades; that is, seismic damages of fill subgrades are light, while seismic damages of cut-and-fill subgrades are obvious.
(2) The mechanical parameters required for the dynamic numerical model can be obtained through cyclic loading and unloading tests. The dynamic mechanical properties of subgrade soil samples are greatly influenced by confining pressure, amplitude, and vibration cycles, and the axial strain of samples increases with the increase in vibration cycles, with the increasing amplitude. Under the same amplitude, more vibration cycles are required for the cumulative strain of soil samples with high confining pressure to reach failure. When the confining pressure remains constant, the greater the amplitude, the greater the axial cumulative deformation of samples, and the faster the cumulative deformation increases.
(3) The 3D dynamic numerical analysis shows that under the same conditions, compared with fill subgrades, cut-and-fill subgrades are more susceptible to earthquakes. Main seismic damages include large plastic strain along the fill-and-cut interface, large deformation extending from the slope top to outside of the slope, and certain bulging trend at the slope toe, all of which finally lead to cracks along the fill-and-cut interface, which is consistent with field investigation results.
(4) Results of existing seismic investigations and shaking table tests show that the interaction between geogrids with reinforcement and soil interface can limit the lateral deformation of subgrade filling, increase the natural vibration frequency of subgrades, make the main frequency of external excitation load far away from the resonance frequency band of subgrades, inhibit the amplification effect of acceleration, and thus reduce the dynamic response amplitude of subgrades. The deformation control method for the subgrades mainly involves geogrids that aim to the control lateral deformation, and geogrids are to be laid on the top of the embankment.

## Figures and Tables

**Table 1:** Information on the damaged highway subgrades.

Chainage	Subgrade Type	Maximum Fill Height	Original Ground Slope Angle	Slope Ratio of the Fill	Reinforcement *	Epicentral Distance/km	Seismic Damage
K32 + 980 –K33 + 030	Cut-and-fill	13	24°	1:1.5–1:1.75	A	7.91	Severe
K33 + 050 –K33 + 100	Cut-and-fill	22.6	36°	1:1.5–1:2.0	A	7.74	Severe
K33 + 940 –K34 + 048	Cut-and-fill	16.3	15°	1:1.5–1:1.75	A	7.51	Mild
K34 + 180 –K34 + 230	Fill	10	9°	1:1.5	B	7.38	Mild
K34 + 435 –K34 + 600	Cut-and-fill	18.8	29°	1:1.5–1:1.75	A	7.25	Severe
K34 + 630 –K34 + 706	Cut-and-fill	12.1	15°	1:1.5–1:1.75	A	7.23	Mild
K35 + 100 –K35 + 150	Fill	12.2	12°	1:1.5–1:1.75	A	6.94	Minor
K35 + 290 –K35 + 430	Fill	19.6	1°	1:1.5–1:1.75	B	6.89	Minor
K35 + 640 –K35 + 760	Fill	21.6	9°	1:1.5–1:2.0	A	6.56	Mild
K35 + 880 –K36 + 050	Fill	21.4	14°	1:1.5–1:2.0	A	6.44	Minor
K36 + 070 –K36 + 100	Cut-and-fill	23.9	36°	1:1.5–1:2.0	A	6.25	Severe
K37 + 025 –K37 + 070	Fill	17.5	3°	1:1.5–1:1.75	A	5.99	Minor
K37 + 840 –K38 + 000	Fill	13.2	2°	1:1.5–1:1.75	A	5.36	Minor
K38 + 550 –K38 + 700	Cut-and-fill	15.6	35°	1:1.5–1:1.75	A	4.53	Severe
K38 + 750 –K39 + 005	Cut-and-fill	12.5	34°	1:1.5–1:1.75	A	4.55	Severe
K39 + 005 –K39 + 100	Fill	10.3	2°	1:1.5–1:1.75	A	4.89	Minor
K42 + 450 –K42 + 620	Fill	13.6	2°	1:1.5–1:1.75	A	5.69	Minor
K43 + 210 –K43 + 310	Fill	19.3	3°	1:1.5–1:1.2	A	5.77	Minor

* A: Diamond-shaped framework structure; B: Retaining wall at slope toe.

**Table 2:** Dynamic mechanical parameters of soil samples.

Vibration Cycles/N	Axial Dynamic Deviator Stress/kPa	Confining Pressure/kPa	Axial Dynamic Stress/kPa	Dynamic Cohesion/kPa	Dynamic Internal Friction Angle/°
10	314.96	50	364.96	23.97	39.19
	381.94	100	481.94		
	635.60	150	785.60		
30	274.54	50	324.54	36.53	33.88
	367.54	100	467.54		
	524.43	150	674.43		
50	257.56	50	307.56	40.15	31.76
	361.02	100	461.02		
	479.58	150	629.58		
80	242.86	50	292.86	42.51	29.96
	355.13	100	455.13		
	441.71	150	591.71		
100	236.18	50	286.18	43.14	29.21
	352.37	100	452.37		
	424.80	150	574.80		

**Table 3:** Material mechanical parameters used in the numerical simulation.

Material	Modulus/kPa	Poisson’s Ratio	Cohesion/kPa	Internal Friction Angle/°
Fill	18,000	0.3	40.15	31.76
Subgrade	100,000	0.28	400	35

## Data Availability

The data presented in this study are available on request from the corresponding author upon reasonable request.
